# Fellow-Workers and the Roll of Honour

**Published:** 1915-10-16

**Authors:** 


### ROUND THE WORLD.

[The Editor invites Early Information and Contributions Marked "Fellow-Workers."]

### Fellow-Workers and the Roll of Honour.

Captain Edward Worrell Carrington, R.A.M.C., killed in recent fighting on the Western front, proceeded to King's College Hospital from Oxford University, and qualified M.R.C.S., L.R.C.P. just two years ago. He joined the Army in the earliest days of the war, and, being sent abroad, received the Military Cross for his services in the field.

Captain Ernest Cotton Deane, R.A.M.C., also killed in the heavy encounters of the past few weeks, had decided upon a military career some time before the war. A Dublin student, he qualified in 1909 by taking the diploma of the Irish Royal Colleges, and two years later entered the R.A.M.C. Subsequently he was sent to India, and before coming to Europe with a draft of the Indian contingent he had been stationed at Lucknow. Captain Deane was always a keen sportsman, and at one time played football for Ireland in International matches. His pluck and gallantry won him the Military Cross before he fell.

Lieutenant Edgar Faulks, R.A.M.C., likewise a fallen hero of the recent advance, left the important post of senior medical officer at Bexley Asylum to take up military work for the time of the war. He qualified from Guy's Hospital in 1902, and subsequently served as house surgeon and in the throat department at that institution; also as clinical assistant at the Hospital for Sick Children in Great Ormond Street. His career in the Army was unfortunately a short one, as he fell in action scarcely three months after taking a commission, and within a few days of reaching the Front.

Lieutenant Kenneth Robinson, R.A.M.C., another medical officer to lose his life in the move forward, left his practice in Llandudno to join the R.A.M.C. some six or seven months ago. A Manchester University student, he qualified M.R.C.S., L.R.C.P. in 1907, and five years later took the M.B., B.S. degrees of London University. Formerly honorary medical officer to the Bramley Cross Girls' Home in Lancashire, he held the appointment of surgeon to the Post Office at Llandudno.

After various changes since the foundation of the institution, the staff of the American Women's War Hospital at Paignton is now constituted as follows: Sir William Osler, honorary consulting physician; Dr. D. Pearce Penhallow, chief surgeon; Dr. F. C. Coller, principal assistant surgeon; Dr. R. P. Borden, Dr. H. M. Goodwin, Dr. Daniel, Dr. H. M. Frost, assistant surgeons; Miss F. E. Bickmore, matron.

Captain W. M. West-Watson, R.A.M.C. (T.F.), who is at present serving with the West Lancashire Casualty Clearing Hospital, has just been appointed one of the honorary assistant physicians to the Bradford Royal Infirmary. Captain West-Watson was a student of Glasgow and Birmingham Universities, qualifying by taking the M.B., B.Ch. of the former in 1903. Subsequently he became an M.D. Glasg., and amongst other successes won the John Hunter Medal in clinical surgery. Formerly he was house surgeon to the Western Infirmary, Glasgow, and house physician to the Cumberland Infirmary, Carlisle.

Lieutenant-Colonel Herbert Lightfoot Eason, R.A.M.C., who has been confirmed in that rank as consulting ophthalmic surgeon to the Mediterranean Expeditionary Force, is one of our leading specialists for diseases of the eye. An M.D., M.S. Lond., he is senior ophthalmic surgeon to Guy's Hospital, and also holds the appointments of honorary ophthalmic surgeon to St. John's Hospital, Lewisham, and to the Jews' Orphanage, West Norwood.

Surgeon Hubert Chitty, R.N.V.R., who has contributed an interesting account of work in a Mediterranean hospital ship to the *British Medical Journal* (October 9, 1915), is in ordinary times assistant surgeon to the Bristol Royal Infirmary. An M.S. Lond., F.R.C.S. Eng., he qualified from University College Hospital in 1904, and his present appointments include those of surgeon to the Cossham Memorial Hospital, and to the Bristol Orthopædic Hospital.

The possible number of medical men available after the war is being steadily reduced, not only by a heavy mortality amongst doctors serving with the R.A.M.C., and by the fact that many young men who in happier days would have joined the hospitals are now in khaki, but also by the deaths amongst students who interrupted their medical course to join combatant ranks. To previous lists of medical students who have given their lives in the service of their country must now be added the following names: Lieutenant A. W. B. Carless, Second-Lieutenant G. F. Dobbin, Lieutenant J. R. Duggan, Second-Lieutenant C. Gillespie, Lieutenant H. A. Munro.

Lieutenant Albert William Buchan Carless was a student of Cambridge and King's College, London, gaining the Warneford entrance scholarship at the latter institution. He was the eldest son of a well-known London surgeon, Professor Albert Carless, F.R.C.S., of King's College and King's College Hospital, who himself holds a commission in the R.A.M.C. (T.F.).

Second-Lieutenant George F. Dobbin and Lieutenant John Rowswell Duggan were both students of Trinity College, Dublin, both lost their lives at the early age of twenty, and both found their resting-place at the Dardanelles.

Whilst serving in France Second-Lieutenant Charles Gillespie received a severe wound in the chest which ultimately proved fatal. A B.Sc. of St. Andrews, he was well advanced with his reading for a medical degree when war broke out, and obtained a commission within a few days. Mr. Gillespie distinguished himself in athletics as well as in science, captaining the athletic and hockey clubs at his university.

The official list of members of the nursing profession who have died on military service contains eight names; they are: E. H. Cole, E. Fearnley, M. H. Johnston, L. M. Swaine, P. A. Pearce, M. A. Walshe, of the Queen Alexandra's Imperial Military Nursing Service; also J. B. Haggard (matron) and F. E. Munroe, of the Canadian Army Nursing Service, both of whom lost their lives in the Mediterranean campaign.

Lieutenant Hugh A. Munro, killed by a bomb, was a fifth-year student of Glasgow University when he took a combatant commission in the Army.

